# PARP Inhibition Shifts Murine Myeloid Cells Toward a More Tolerogenic Profile In Vivo

**DOI:** 10.3390/biom15081149

**Published:** 2025-08-09

**Authors:** Jose R. Pittaluga-Villarreal, Casey M. Daniels, Tara Capece, Pauline R. Kaplan, Martin Meier-Schellersheim, Aleksandra Nita-Lazar

**Affiliations:** 1 Functional Cellular Networks Section, Laboratory of Immune System Biology, National Institute of Allergy and Infectious Diseases (NIAID), National Institutes of Health (NIH), Bethesda, MD 20892-1892, USA; jose.pittalugavillarreal@nih.gov (J.R.P.-V.); cs.dnls@gmail.com (C.M.D.); prkaplan2017@gmail.com (P.R.K.); 2 Computational Biology Section, Laboratory of Immune System Biology, National Institute of Allergy and Infectious Diseases (NIAID), National Institutes of Health (NIH), Bethesda, MD 20892-1892, USA; tara.capece@gmail.com (T.C.); mms@niaid.nih.gov (M.M.-S.)

**Keywords:** olaparib, PARP inhibitors, myeloid cells, dendritic cells, neutrophils, macrophages, immunosuppression, N2 neutrophils, M2 macrophages, immunotherapy

## Abstract

The human Poly ADP-ribose Polymerase (PARP) family comprises 17 enzymes responsible for the transfer of ADP-ribose to proteins, forming poly- or mono-ADP-ribosylation. This post-translational modification regulates DNA repair and programmed cell death, processes affecting cancer biology. PARP inhibitors, including the FDA-approved olaparib, are used to treat BRCA-dependent breast and ovarian cancers. Although therapies with use of PARP inhibitors are showing clinical success, their effects on the immune system remain understudied. Prior work has shown that PARP inhibition can modulate inflammatory responses and alter innate immunity. In this study, we evaluated the immunomodulatory effects of olaparib on myeloid cells in vivo, focusing on bone marrow and spleen. Olaparib treatment altered the composition and activation state of dendritic cells, neutrophils, and macrophages. In the bone marrow, olaparib increased the proportion of cDC2 population, mature neutrophils and inflammatory macrophages expressing CD80. In contrast, splenic myeloid cells exhibited enhanced expression of markers associated with tolerogenic phenotypes, including CD206 and CD124 in neutrophils and macrophages. The spleen also showed an increase in immature monocyte-derived dendritic cells (CD206+) and a bias toward the cDC2 subset. These findings indicate that PARP inhibition can induce short-term phenotypic remodeling of myeloid cell populations, promoting a more immunoregulatory profile, especially in the spleen. These changes may contribute to an altered immune landscape with implications for anti-tumor immunity.

## 1. Introduction

Poly ADP-ribose Polymerases (PARPs) constitute a family of 17 enzymes responsible for the ADP-ribosylation of proteins, a wide-spread post-translational protein modification involved in the regulation of essential cellular processes, including DNA repair and programmed cell death, both of which are highly relevant to cancer biology [1]. The therapeutic potential of targeting PARPs has led to the development and FDA approval of several PARP inhibitors (PARPi), such as olaparib, which are currently used in the treatment of BRCA-mutation-dependent breast, ovarian, and prostate cancers due to their ability to induce synthetic lethality in tumor cells [2,3].

Although the antitumor efficacy of PARP inhibitors is well established, their effects on the immune system, particularly on immune cell populations, remain incompletely understood. Recent studies have shown that PARP inhibition may alter antigen presentation, as indicated by increased MHC-II [4] and a reverse correlation between ADP-ribosylation the immune checkpoint ligand, PD-L1 [5]. Jiao et al. further demonstrated that pharmacologic inhibition of PARP1 leads to upregulation of PD-L1 in tumor cells, suggesting an immunomodulatory axis involving ADP-ribosylation and immune escape mechanisms [6].

Even less is known about the impact of PARP inhibitors on myeloid cell subsets, despite clinical observations indicating olaparib-associated myelosuppression [7,8]. In a previous work, we have shown that ADP-ribosylation status of many proteins changes during the inflammatory response and affects the innate immune pathways. PARP inhibitors affected these changes and increased survival of LPS-treated mice [9], suggesting that PARP inhibition has an effect on the myeloid cell population.

Given that myeloid cells -mainly dendritic cells (DC), neutrophils (PMN), and macrophages (Mac)-play crucial roles in both inflammation and cancer progression, and that their functional polarization can shape the immune contexture of the tumor microenvironment (TME) [10] and many clinical studies demonstrated that tumor-associated macrophages and neutrophils affect the therapeutic outcome [11,12,13], it is critical to understand how PARP inhibition may impact these populations.

In this study, we aimed to evaluate whether olaparib treatment affects the composition or activation state of myeloid cells in vivo. We hypothesized that PARP inhibition could shift these cells toward more immunoregulatory phenotypes, potentially diminishing inflammatory or antitumor responses. To evaluate this, mice were treated systemically with olaparib, and immune cells were subsequently isolated from primary (bone marrow) and secondary (spleen) lymphoid organs. We employed multiparametric flow cytometry to characterize potential phenotypic and functional shifts in dendritic cells, neutrophils, monocytes, and macrophages.

Our findings suggest that systemic treatment with olaparib alters the functional profile of myeloid cells in vivo, favoring a more immunoregulatory profile in the spleen.

## 2. Materials and Methods

### 2.1. Mice

Female C57BL/6J mice aged 12 to 16 weeks were obtained from Jackson Laboratories and maintained in specific pathogen-free conditions at an American Association for Laboratory Animal Care-accredited animal facility at the National Institute of Allergy and Infectious Diseases (NIAID) under study protocol LISB-17E approved by the NIAID Animal Care and Use Committee following the guidelines of the Office of Animal Care and Use (OACU). Mice were maintained under a 12 h light–dark cycle at 22 ± 2 °C and fed with standard diet and water ad libitum.

### 2.2. PARP Inhibitor Treatments

Olaparib (Cat#: S1060 Batch: S106024, Selleck Chemicals, Houston, TX, USA) treated mice were injected intraperitoneally (50 mg/kg) and control mice were injected with vehicle: 10% (2-hydroxypropyl)-β-cyclodextrin [HPβCD] (Cat#:H-107, Sigma Aldrich, St. Louis, MO, USA), 10% DMSO, 1x PBS. The intraperitoneal injections were performed on day 0, day 3 and day 5. At day 6 mice were euthanized with isoflurane followed by cervical dislocation.

### 2.3. Sample Preparation

Bone marrow (BM) and spleen were collected from euthanized mice on day 6 post-treatment. BM cells were flushed from femurs and tibias using cold PBS supplemented with 2% fetal bovine serum (FBS) and then filtered with a 70 µm cell strainer. Spleens were mechanically disaggregated and passed also through a 70 µm cell strainer. Red blood cells were lysed using eBiocience™ RBC Lysis Buffer (Multi-species) (Thermo Fisher, Waltham, MA, USA, 00-4300-54) for 5 min at room temperature, followed by washing and resuspension in FACS buffer (PBS + 2% FBS + 2 mM EDTA). For surface staining, 2 × 10^6^ cells were incubated with fluorochrome-conjugated antibodies (Table S1) for 30 min at 4 °C in the dark.

### 2.4. Data Acquisition and Analysis

**Figure 1:**
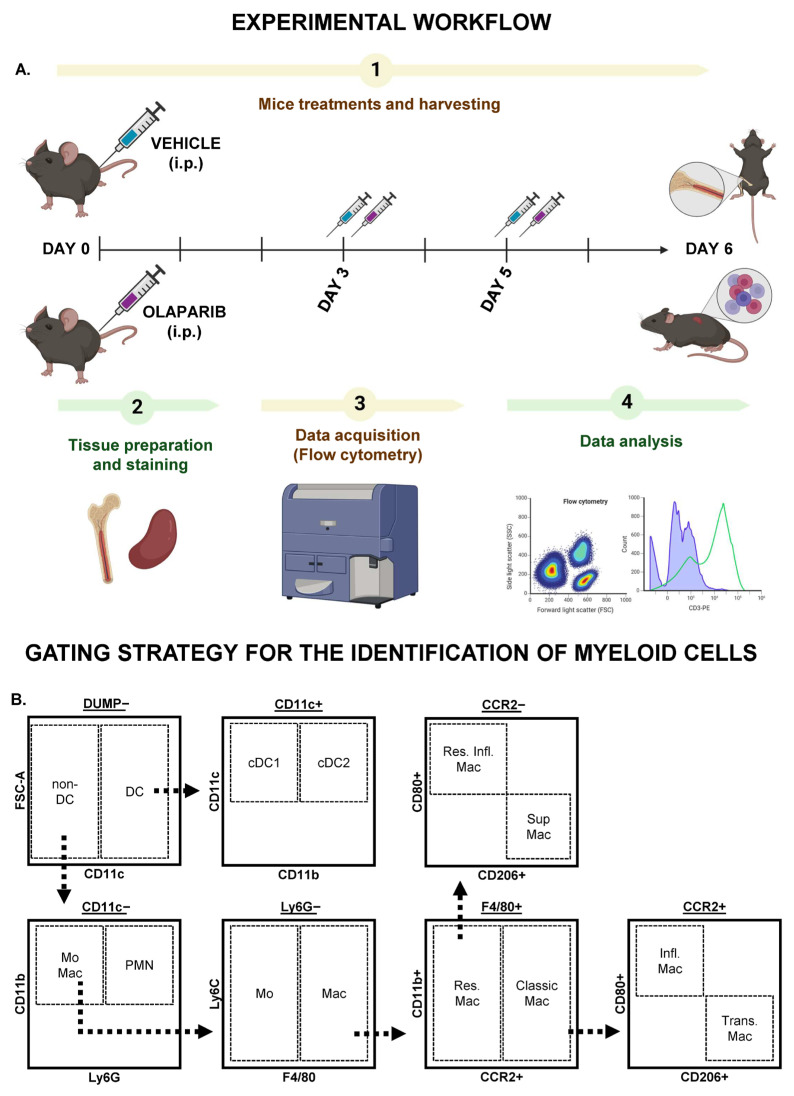
Experimental and data analysis workflow. (**A**) Mice received intraperitoneal injections of vehicle or olaparib on Day 0, with subsequent injections on Days 3 and 5. On Day 6, the mice were euthanized, and bone marrow (BM) and spleens were collected. Samples were then prepared and stained for flow cytometry acquisition, followed by data analysis. (**B**) After excluding non-myeloid cells (T cells, NK cells, B cells, and erythroid lineage), CD11c^+^ cells were classified as dendritic cells (DC), with further subdivision into conventional type 1 or type 2 DC (cDC1 and cDC2). The CD11c^−^ population was further classified into neutrophils (PMN, CD11b^+^Ly6G^+^), and CD11b^+^Ly6G^−^ cells were classified as monocytes (Mo, F4/80^−^). Macrophages (Mac, F4/80^+^) were further categorized into CCR2^−^ (resident Mac) and CCR2^+^ (classical Mac), with subsequent classification into inflammatory Mac (CCR2^+^CD80^+^), transitional Mac (CCR2^+^CD206^+^), resident inflammatory Mac (CCR2^−^CD80^+^), and suppressor Mac (CCR2^−^CD80^+^).

After staining, cells were washed, resuspended in FACS buffer, and acquired on a BD FACSSymmphony™ flow cytometer (BD Biosciences, San Jose, CA, USA). Data from flow cytometry was analyzed using FlowJo™ v10.8 Software (BD Life Sciences, Franklin Lakes, NJ, USA). During the study, single cells expressing CD45R (B cells), CD3ε (T cells), CD335 (NK cells), and TER-119 (erythroid lineage cells) were considered as DUMP and were excluded from the analysis. The remaining cells were considered as cells of interest (COI) (Figure S1). Dendritic cells (DC) were classified as cells CD11c^+^, the CD11c^−^ cells were further divided as cells CD11b^+^Ly6G^+^ as neutrophils (PMN) and CD11b^+^Ly6G^−^ as monocytes (Mo) and macrophages (Mac). The latter were classified according to the expression of F4/80, a characteristic marker present in Mac [14,15] (Figure 1B). The complete gating strategy shown directly from dot plots is depicted in Supplementary Materials (Figure S1).

### 2.5. Statistical Analysis

All statistical analyses were performed using GraphPad Prism (v10.0). Data are presented as mean ± standard error of the mean (SEM), with each dot representing a biological replicate (individual mouse). Normality of data distribution was assessed using the Shapiro–Wilk test. Comparisons between groups (vehicle vs. olaparib) were performed using unpaired two-tailed Student’s *t*-test for normally distributed data, or Mann–Whitney *U* test for non-parametric data. *p*-values < 0.05 were considered statistically significant.

## 3. Results

### 3.1. Olaparib Alters DC Subsets in Bone Marrow and Spleen

Mice were treated with olaparib or vehicle for up to 6 days, bone marrow (BM) and spleen were collected for further phenotyping staining (Table S1), acquisition by flow cytometry and subsequent software analysis (Figure 1A). No overt clinical signs of distress, weight loss, or changes in overall appearance were observed in either group. Gross examination of organs at necropsy did not reveal any size differences or signs of inflammation.

**Figure 2:**
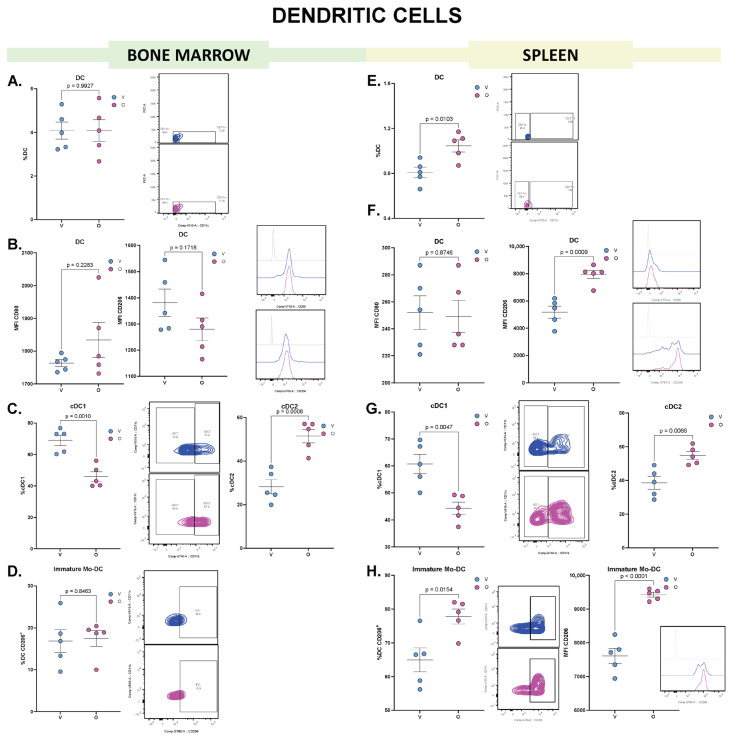
Flow cytometry analysis of DC from murine BM and spleen reveals distinct phenotypic changes following olaparib treatment. (**A**) Frequency of DC in BM following vehicle (V, blue) or olaparib (O, pink) treatment. Dot plots show the percentage of DC within the gated cells of interest (COI^+^); a representative dot plot is shown to the right. (**B**) Median fluorescence intensity (MFI) of CD80 and CD206 in BM-derived DC; representative histograms are shown to the right. (**C**) Frequency of conventional type 1 and type 2 DC subsets (cDC1: CD11^low^. cDC2: CD11^high^) within the DC populations; a representative dot plot is shown in the middle. (**D**) Frequency of immature Mo-derived DC (DC CD206^+^) within the DC population; a representative dot plot is shown to the right. (**E**) Frequency of DC in spleen, dot plots show DC frequency within the COI^+^ population; a representative dot plot shown to the right. (**F**) MFI of CD80 and CD206 in splenic DC; representative histograms are shown to the right (**G**) Frequency of cDC1 and cDC2 subsets within splenic DC; representative dot plot is shown in the middle. (**H**) Frequency of DC CD206^+^ within total splenic DC, and MFI of CD206 within the population; representative dot plot and histogram are shown to the right. Each dot represents an individual biological replicate; horizontal lines indicate mean ± SEM (*n* = 5). Statistical analysis: comparisons were performed using unpaired two-tailed Student’s *t*-test (for normally distributed data) or Mann–Whitney *U* test (for non-parametric data).

The first myeloid population we focused on were DC, immune cells that boost immune responses by presenting antigens on their surface to other cells of the immune system and play a crucial role in adaptive immunity. Olaparib did not alter murine DC proportion in BM (Figure 2A), and MFI of CD80 and CD206 expression in the general DC population were not statistically different between treatments (Figure 2B). Further subclassification of DC into conventional type 1 and type 2 DC (cDC1, cDC2) by their CD11b expression (CD11b^low^: cDC1, CD11b^high^: cDC2) [16,17] demonstrated that olaparib induced a decrease in the proportion of cDC1 in BM and subsequently an increase in cDC2 (Figure 2C). This DC subset is involved in the Th2 responses, which are more tolerogenic [18]. There were no differences in the frequency of DC CD206^+^, a marker associated with immature monocyte-derived DC [19] (Figure 2D).

In contrast to BM, splenic DC were increased after olaparib treatment (Figure 2E) along with upregulation of the CD206 marker (Figure 2F). This mannose receptor (CD206) supports a role in Th2 polarization [20]. The MFI of CD80 was not altered. In the same way that BM, splenic cDC1 were lower in olaparib treatment than in control (vehicle) accompanied by an increase in the percentage of cDC2, respectively (Figure 2G). In addition, the proportion of DC CD206^+^ cells were increased in mice that received the olaparib treatment, along with an increase in the marker expression on the DC surface (Figure 2H).

These results indicate that olaparib induced a change in the DC subpopulations in the BM and spleen, favoring an increase in cDC2 and DC CD206^+^ subsets and the DC markers indicating a more tolerogenic response.

### 3.2. Olaparib Changes the Phenotype of BM-Derived and Splenic PMN

**Figure 3:**
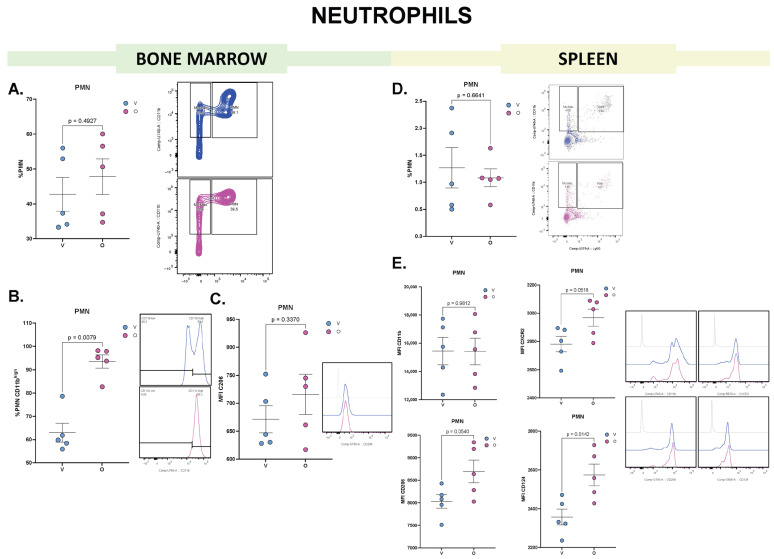
Flow cytometry analysis of PMN from BM and spleen reveals distinct phenotypic changes following olaparib treatment. (**A**) Frequency of PMN in BM following vehicle (V, blue) or olaparib (O, pink) treatment. Dot plots show the percentage of PMN within the gated cells of interest (COI^+^); a representative dot plot is shown to the right. (**B**) Frequency of mature PMN (CD11b^high^) within the PMN population; a representative histogram is shown to the right. (**C**) MFI of CD206 in BM-derived PMN; a representative histogram is shown to the right. (**D**) Frequency of PMN in spleen, dot plots show PMN frequency within the COI^+^ population; a representative dot plot is shown to the right. (**E**) MFI of CD11b, CXCR2, CD206 and CD124 in splenic PMN; representative histograms are shown to the right. Each dot represents an individual biological replicate; horizontal lines indicate mean ± SEM (*n* = 5). Statistical analysis: comparisons were performed using unpaired two-tailed Student’s *t*-test (for normally distributed data) or Mann–Whitney *U* test (for non-parametric data).

PMN are myeloid cells generated in the BM which are part of the first line of defense against infections and inflammation, playing a crucial role in innate immune responses. Olaparib treatment in mice did not alter murine PMN population in the BM (Figure 3A). Although mice treated with the drug showed an increase in the proportion of mature PMN (Figure 3B), the MFI of CD206 expression in the general PMN population was not statistically different between treatments (Figure 3C), Notably, this marker is typically upregulated in PMN associated with immunosuppressive or tolerogenic environment [18].

When this myeloid population was evaluated in the spleen, we observed that total splenic PMN numbers were not altered between treatments (Figure 3D). Nonetheless, MFI measurements of markers associated with recruitment (CXCR2), and immunosuppression (CD124, CD206) revealed distinct changes (Figure 3E). CD11b expression was not significantly different between treatments, but CXCR2 and CD206 showed a trend toward upregulation in splenic PMN from olaparib-treated mice. Importantly, CD124 was significantly increased in this group.

These results indicate that olaparib promotes PMN maturation in the BM and enhances the expression of surface markers associated with recruitment and immunosuppressive functions in splenic PMN, suggesting a more tolerogenic PMN phenotype in the spleen.

### 3.3. Exposure to Olaparib Did Not Affect Total Mo Population but Altered the Distribution of Mac in BM

Mo are leukocytes produced in the BM that circulate in the blood and can differentiate into Mac or DC in tissues, playing a key role in the immune system. The Mo population from BM and spleen of olaparib-treated mice showed no statistically significant differences in the proportion of total Mo compared to vehicle-treated mice in either tissue (Figure S2A,D). Similarly, no changes were observed in the frequencies of Mo subsets, including classical Mo (Ly6C^+^CCR2^+^) and patrolling Mo (Ly6C^−^CCR2^−^) [21] (Figure S2B,C,E). Notably, a trend toward increased frequency of patrolling Mo was observed in the spleen following olaparib treatment, although this change did not reach statistical significance (Figure S2F).

**Figure 4:**
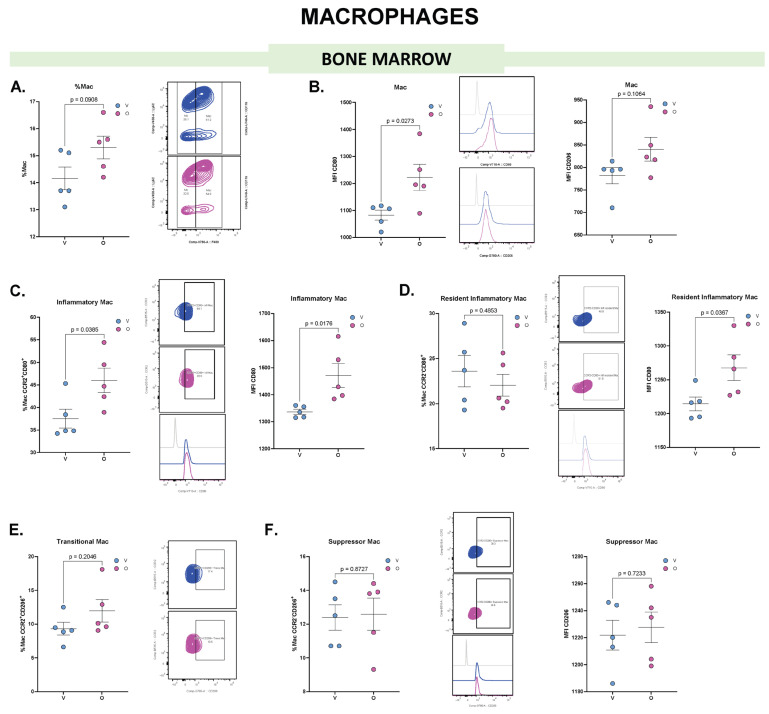
Flow cytometry analysis of Mac from murine BM reveals distinct phenotypic changes following olaparib treatment. (**A**) Frequency of Mac in BM following vehicle (V, blue) or olaparib (O, pink) treatment. Dot plots show the percentage of Mac within the gated cells of interest (COI^+^); a representative dot plot is shown to the right. (**B**) MFI of CD80 and CD206 in BM-derived Mac; representative histograms are shown in the middle. (**C**) Frequency of inflammatory Mac (Ly6C^+^CCR2^+^CD80^+^) within total Mac, and MFI of CD80 in the inflammatory Mac population; representative dot plot and histogram are shown in the middle. (**D**) Frequency of resident inflammatory Mac (Ly6C^+^CCR2^−^CD80^+^) within total Mac, and MFI of CD80 in the resident inflammatory Mac population; representative dot plot and histogram are shown in the middle. (**E**) Frequency of transitional Mac (Ly6C^+^CCR2^+^CD206^+^) within total Mac; a representative dot plot is shown to the right. (**F**) Frequency of suppressor Mac (Ly6C^+^CCR2^−^CD80^+^) within total Mac, and MFI of CD206 in the suppressor mac population; representative dot plot and histogram are shown in the middle. Each dot represents an individual biological replicate; horizontal lines indicate mean ± SEM (*n* = 5). Statistical analysis: comparisons were performed using unpaired two-tailed Student’s *t*-test (for normally distributed data) or Mann–Whitney *U* test (for non-parametric data).

Mac are professional phagocytes specialized in removing dying or dead cells, and microorganisms, playing roles in both innate and adaptive immune responses. They can also sense and respond to infection and tissue damage, contributing to a pro-inflammatory response or tissue repair and regeneration. In olaparib-treated mice, no alteration was observed in the absolute frequency of the Mac population in BM (Figure 4A). However, a significant increase in the MFI of CD80, a marker associated with enhanced antigen presentation, was noted in the olaparib-treated mice (Figure 4B). Although there was a trend toward increased CD206, a marker related to more tolerogenic or immunosuppressive effects, this increase was not statistically significant (Figure 4B).

Mac can be classified based on their ontogeny and activation status. Mo-derived CCR2^+^ Mac are typically recruited to tissues in response to inflammation or injury, while CCR2^−^ Mac are generally considered tissue-resident, associated with local proliferation, tissue regeneration and immunoregulatory functions [22,23]. Additionally, Mac may adopt different activation states: pro-inflammatory (M1-like) Mac express CD80, whereas tolerogenic (M2-like) Mac are characterized by CD206 expression [24,25]. In our study, we classified Mac expressing CCR2^+^CD80^+^ as inflammatory Mac, and we observed an increase in their population in the BM of mice treated with olaparib, accompanied by an increase in the MFI of CD80 in this subpopulation (Figure 4C). In contrast, resident Mac with inflammatory features (CCR2^−^CD80^+^) did not change the population frequency but exhibited an increase in the MFI of CD80 (Figure 4D). On the other hand, Mac co-expressing CCR2^+^ and CD206^+^ were classified as transitional Mac, while those lacking CCR2 but expressing CD206 (CCR2^−^CD206^+^) were categorized as suppressor (M2-like) Mac. Neither subpopulation showed differences in their population frequencies (Figure 4E,F), nor in the MFI of CD206 in suppressor Mac.

These results suggest that olaparib promotes a more stimulatory and pro-inflammatory profile in the BM, due to the increase in Mac subsets with markers associated with an M1-like response.

### 3.4. Olaparib Increases Splenic Mac CD206^+^ Subpopulation Associated with the Tolerogenic Response

**Figure 5:**
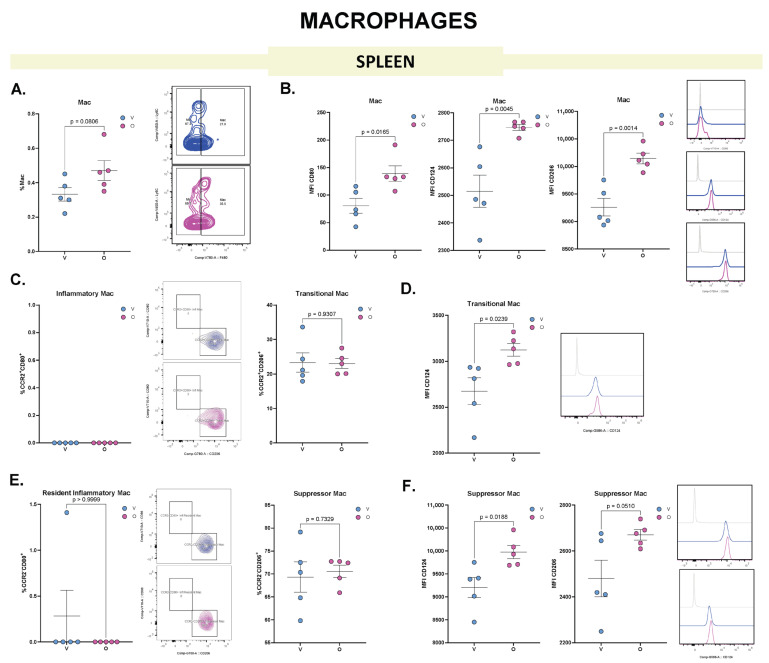
Flow cytometry analysis of Mac from murine spleen reveals distinct phenotypic changes following olaparib treatment. (**A**) Frequency of Mac in spleen following vehicle (V, blue) or olaparib (O, pink) treatment. Dot plots show the percentage of Mac within the gated cells of interest (COI^+^); a representative dot plot is shown to the right. (**B**) MFI of CD80, CD124 and CD206 in splenic Mac; representative histograms are shown to the right. (**C**) Frequency of inflammatory Mac (Ly6C^+^CCR2^+^CD80^+^) and transitional Mac (Ly6C^+^CCR2^+^CD206^+^) within total Mac; representative dot plots are shown in the middle. (**D**) MFI of CD124 in the transitional macrophage population; representative histogram is shown to the right. (**E**) Frequency of resident inflammatory Mac (Ly6C^+^CCR2^−^CD80^+^) and suppressor Mac (Ly6C^+^CCR2^−^CD80^+^) within total Mac; representative dot plot and histogram are shown in the middle. (**F**) MFI of CD124 and CD206 in the suppressor Mac population; representative histograms are shown to the right. Each dot represents an individual biological replicate; horizontal lines indicate mean ± SEM (n = 5). Statistical analysis: comparisons were performed using unpaired two-tailed Student’s *t*-test (for normally distributed data) or Mann–Whitney *U* test (for non-parametric data).

Evaluation of splenic Mac following olaparib treatment revealed a non-significant trend toward increased Mac frequency (Figure 5A). However, the MFI of CD80, IL-4 receptor alpha (CD124) and CD206 were significantly elevated (Figure 5B).

In the subpopulation analysis, splenic inflammatory Mac were not detected. Transitional Mac were present without any significant change in frequency (Figure 5C); nonetheless, their expression of CD124 was significantly increased in olaparib-treated mice (Figure 5D). Among resident Mac subsets, resident inflammatory Mac were detected, and the frequency of suppressor Mac remained unchanged by treatment (Figure 5E), although this subset exhibited a significant increase in the MFI of both CD124 and CD206.

These findings suggest that olaparib treatment does not substantially alter the distribution of splenic Mac subpopulations, but it induces the upregulation of markers associated with a more immunoregulatory or suppressive phenotype.

## 4. Discussion

Previous studies have reported adverse effects associated with PARP inhibitors, including an increase in myelodysplastic syndromes and acute myeloid leukemia [26]. Additionally, olaparib has been shown to enhance pro-tumor features of Mac within the TME by decreasing glycolysis and shifting metabolism towards lipid pathways in breast cancer models [27]. In our study, systemic administration of olaparib in mice modulated the composition and phenotype of myeloid cells in the BM and spleen, particularly affecting DC, PMN and Mac.

Olaparib treatment led to an increased population of cDC2 in both organs. This subset is associated with Th2 responses [28]. Furthermore, phenotypic markers indicative of a tolerogenic response, such as CD206 and CD124, were upregulated in splenic PMN and Mac. These changes suggest a shift towards an immunomodulatory profile that could impair anti-tumor immunity within the TME [11,29].

In the spleen, olaparib-treated mice exhibited an increase in DC population compared to the control mice. Both splenic and BM-derived DC showed a higher percentage of cDC2, a subset implicated in the induction of Th17 and Th2 immune responses to extracellular pathogens, parasites, and allergens [28]. Also, cDC2 have been associated with both anti- and pro-tumoral immune responses [30]. Notably, splenic DC from olaparib-treated mice demonstrated elevated expression of CD206, a marker associated with immature monocyte-derived DC [19]. Immature DC have been reported to enhance tumor proliferation in breast cancer [31] and were shown to be strongly biased towards induction of functional Tregs that contribute to the maintenance of a pro-tumoral immune status [32]. Moreover, CD206 expression in DC has been linked to Th2 responses, as evidenced by studies showing that CD206 knockout mice exhibit enhanced pro-inflammatory profiles [20]. Further studies may be performed using a broader spectrum of markers to properly identify all DC subsets [33].

Our study revealed an increase in mature PMN in the BM of olaparib-treated mice. This observation aligns with previous reports indicating that olaparib preferentially targets myeloid cells, leading to a reduction in blasts and promyelocytes and an increase in mature granulocytes [34]. Although we did not observe an overall increase in the PMN population, splenic PMN from olaparib-treated mice had elevated surface expression of CXCR2, a chemokine receptor critical for PMN migration [35]. High CXCR2 expression has been associated with poor prognosis in various cancers, including acute myeloid leukemia, breast cancer, and ovarian cancer, due to its role in promoting tumor cell proliferation and metastasis [36]. Yu et al. demonstrated that recruited CXCR2^+^ PMN were responsible for the pro-metastatic effects in mesenchymal stromal cells since chemotaxis inhibition abolished PMN recruitment and tumor metastasis [37]. Furthermore, PMN from olaparib-treated mice showed increased expression of CD206 and CD124 on their surface. The upregulation of CD206 in PMN suggests a shift towards an N2 phenotype, characterized by immunosuppressive functions supporting tumor growth [38,39]. Similarly, CD124, the alpha chain of the IL-4 receptor, is indicative of sensitivity to IL-4, a cytokine prevalent in Th2-type responses. The presence of IL-4 can drive PMN towards an N2 phenotype, further contributing to an immunoregulatory environment [38,40]. These findings suggest that olaparib not only influences PMN maturation in the BM but also modulates their functional phenotype in the spleen, potentially promoting an immunosuppressive environment that could impair anti-tumor immunity.

Since PARP-1 is an enzyme linked to the recognition of DNA damage and the recruitment of the DNA repair machinery, its inhibition is associated with an increased risk of genotoxic effects and there are possible long-term treatment side effects. PARP-1 inhibition results in a reduction in NF-κB activation and inflammatory cytokine expression [41]. Anti-inflammatory effects of PARP-1 inhibitor were also observed by Delinois et al., who demonstrated that PARP inhibition in LPS-stimulated PMN abrogated inflammasome-mediated NET formation [42].

In the spleen, we found an increase in Mac with higher surface expression of CD124 and CD206, suggesting that olaparib could shift Mac towards an M2 profile. Phenotypically, immunosuppressive M2 Mac are frequently involved in blocking the natural immune response against tumors. Selective depletion of these pro-tumor immune cells and administration of cytokine inhibitors have been shown to reduce the suppressive nature of the TME [43]. On the other hand, olaparib induced an increase in CD80 surface markers in BM Mac, including resident and non-resident inflammatory Mac, indicating an activation state in the BM. Wang et al. reported that olaparib causes reprogramming of tumor-associated macrophages (TAMs) toward higher cytotoxicity and phagocytosis in BRCA1-related breast cancer [44].

The contrasting effects of olaparib on Mac phenotypes in the BM and spleen, as well as those reported in different tumors, may reflect the distinct microenvironmental contexts of these organs. The BM, being a primary hematopoietic site, may favor the activation of Mac towards an M1-like phenotype upon DNA damage-induced stress. In contrast, the spleen, as a secondary lymphoid organ involved in immune regulation, may promote the differentiation of Mac towards an M2-like phenotype in response to systemic signals. These findings underscore the complexity of the immunomodulatory role of PARP inhibitors and highlight the importance of considering tissue-specific contexts when evaluating their effects on immune cell populations.

Our study focused on the primary (BM) and secondary (spleen) lymphoid organs to characterize tissue-resident myeloid populations. While blood and lymph nodes would provide complementary insights, particularly regarding trafficking and systemic immune modulation, they were not analyzed in this short-term treatment model. Consequently, further research is needed to assess the effects of olaparib within a tumor-bearing context, these results should be interpreted with caution and validated in tumor-bearing models, as the immune landscape in tumors can markedly alter drug responses. Moreover, our analyses were limited to phenotypic assessments, in this study we have not considered the cytokines/chemokines in the microenvironment, which is known to be significant in humans [45] thus, incorporating functional assays, particularly those evaluating immunometabolism parameters and a broader cytokine profiling, would be essential to elucidate the functional implications of the observed phenotypic alterations. Additionally, the current study examined only the short-term effects of olaparib; therefore, future studies should consider evaluating the mid- to long-term impacts of the drug on immune cell dynamics and considering age-related changes in myelopoiesis and immune cell composition that may become more prominent in older mice [46].

The induction of tolerogenic myeloid features by olaparib, whose mechanism of action in tumors is not inducing immunogenicity, but preventing the PARP-mediated DNA repair, may indeed appear counterintuitive in the context of cancer therapy. However, similar observations have been reported in tumor-bearing models. For example, Mehta et al. showed that PARP inhibitors can enhance both anti- and pro-tumor features of macrophages via glucose and lipid metabolic reprogramming, mediated by the sterol regulatory element-binding protein 1 (SREBP-1) pathway [27]. In contrast, Wang et al. demonstrated that olaparib reprograms macrophages in the tumor microenvironment toward a more cytotoxic, anti-tumor phenotype [44]. Notably, both studies used the same dose as ours (50 mg/kg i.p.). Nonetheless, we acknowledge that species-specific immune differences and the absence of tumors in our model limit the direct extrapolation of our findings to the tumor context.

Our study demonstrates that systemic administration of olaparib in mice induces a shift towards a more tolerogenic or immunoregulatory profile within myeloid cell populations, particularly affecting DC, splenic PMN, and Mac in spleen. These phenotypic alterations are associated with pro-tumoral immune responses observed in certain cancer models and may compromise the anti-tumor immune environment, potentially diminishing the therapeutic efficacy of olaparib.

## 5. Conclusions

Olaparib, a PARP inhibitor, induces changes in myeloid populations in the BM and spleen, favoring an increase in immature monocyte-derived DC, recruitment of PMNs in the spleen, along with features consistent with an N2-like phenotype, and an increase in M2-like Mac. These immunomodulatory effects may contribute to a suppressive TME, potentially impacting the therapeutic efficacy of the drug.

## Data Availability

The flow cytometry primary data have been retained and are available from the authors upon request.

## Supplementary Materials

The following supporting information can be downloaded at: https://www.mdpi.com/article/10.3390/biom15081149/s1, Figure S1: Flow cytometry gating strategy for myeloid subset populations.; Figure S2: Flow cytometry analysis of Mo from BM and spleen did not reveal any distinct phenotypic changes following olaparib treatment.; Table S1: Antibodies.

## Abbreviations

The following abbreviations are used in this manuscript:

PARPs	Poly ADP-ribose Polymerases
TME	Tumor microenvironment
BM	Bone marrow
DC	Dendritic cells
PMN	Polymorphonuclear neutrophils/Neutrophils
Mac	Macrophages
Mo	Monocytes
COI	Cell of interest
cDC1	conventional dendritic cells type 1
cDC2	conventional dendritic cells type 2
