# Supplementary figures and images for: PARP Inhibition Shifts Murine Myeloid Cells Toward a More Tolerogenic Profile In Vivo
Source: Biomolecules. 2025 Aug 9;15(8):1149. doi: 10.3390/biom15081149 (PMC12384135; doi:10.3390/biom15081149)

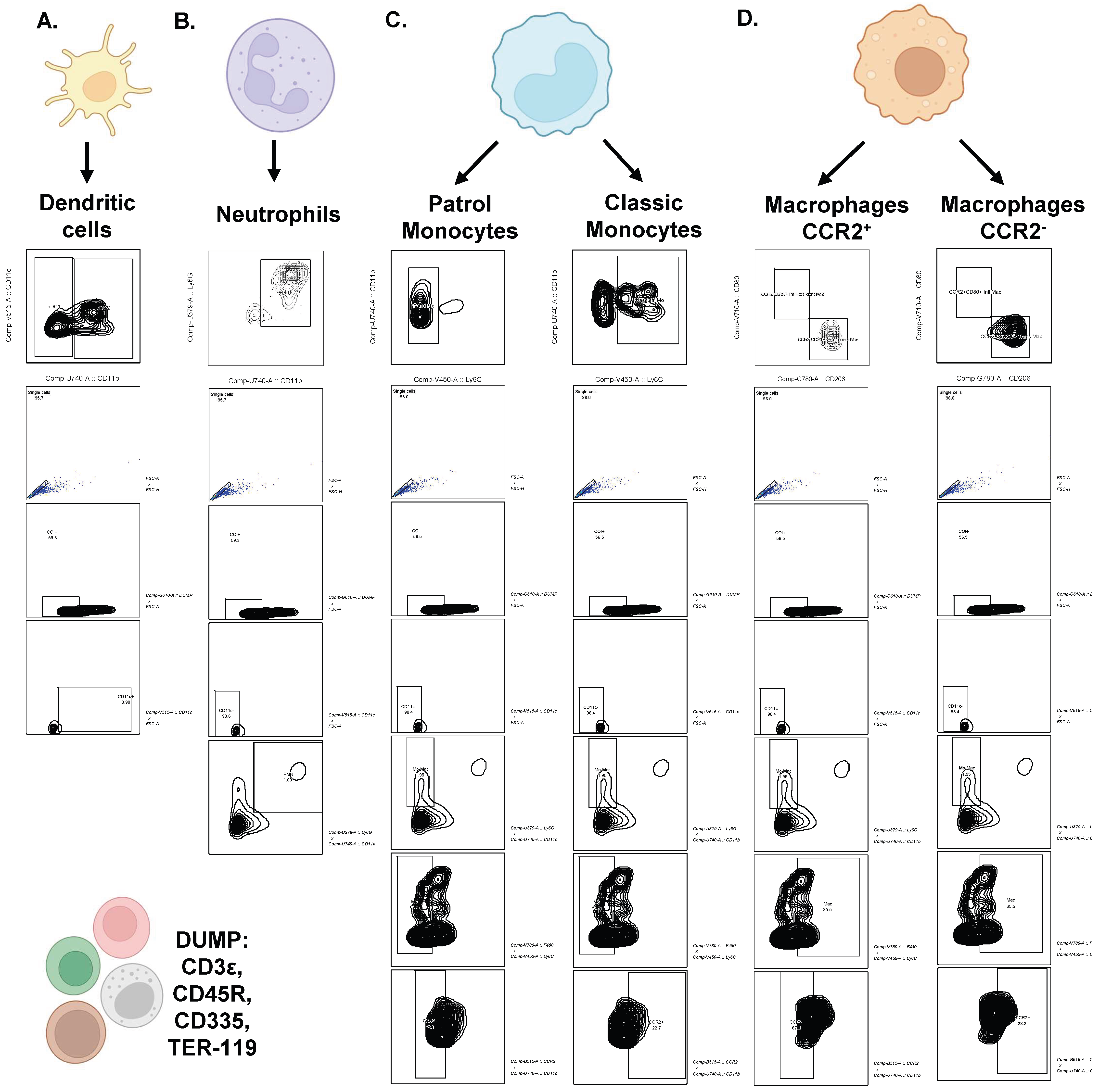

Supplement: Supplementary file 1 [file biomolecules-15-01149-s001.zip › biomolecules-3727212_FigS1.png]

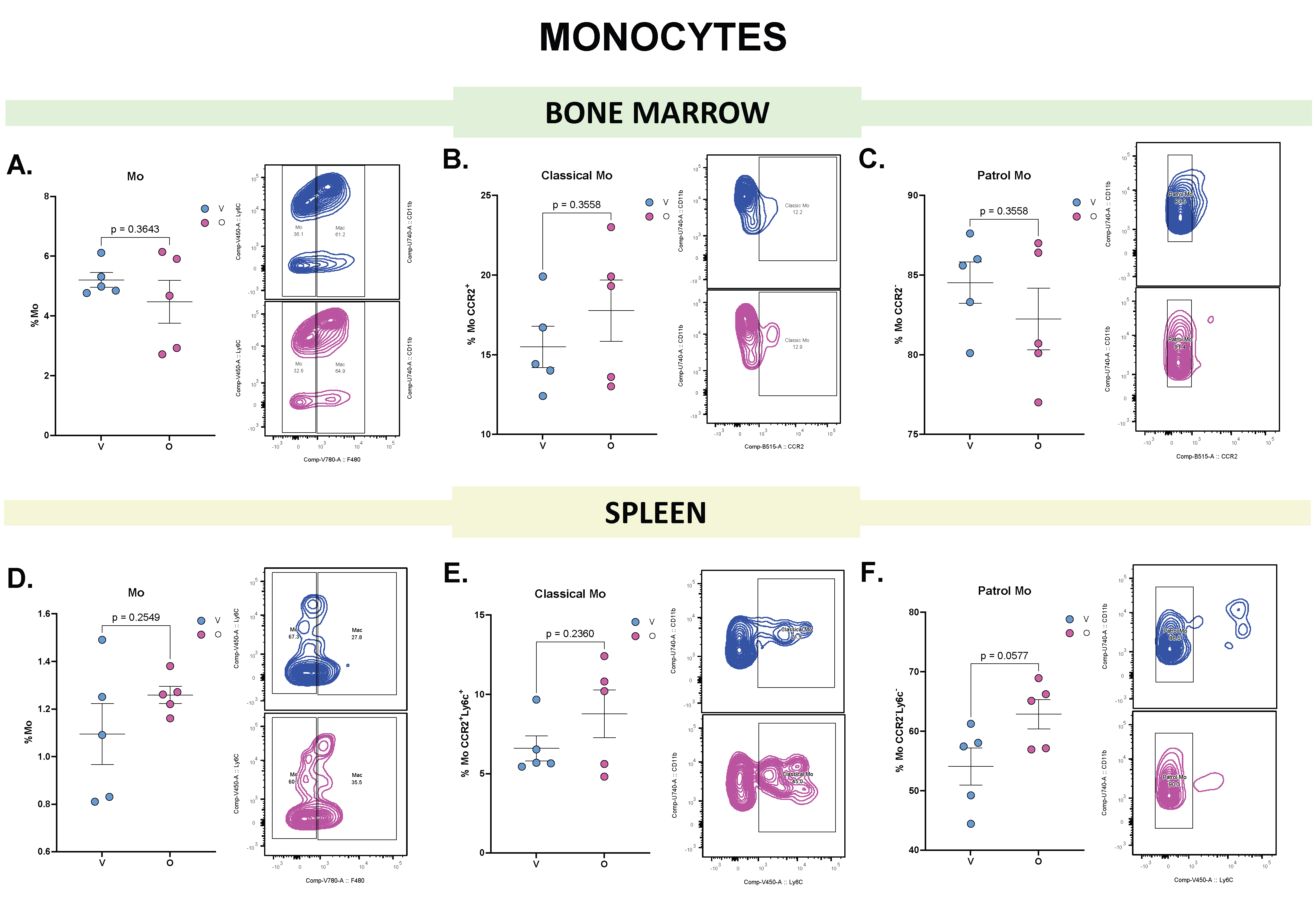

Supplement: Supplementary file 1 [file biomolecules-15-01149-s001.zip › biomolecules-3727212_FigS2.png]
